# Shape Memory Polyurethane Materials Containing Ferromagnetic Iron Oxide and Graphene Nanoplatelets

**DOI:** 10.3390/ma10091083

**Published:** 2017-09-14

**Authors:** Magdalena Urban, Michał Strankowski

**Affiliations:** Polymer Technology Department, Chemical Faculty, Gdańsk University of Technology, 11/12 Narutowicza street, 80233 Gdańsk, Poland; mhburban@gmail.com

**Keywords:** polyurethanes, memory shape, graphene nanoplates, ferromagnetic iron oxide, thermal properties, polymer composites, rheological properties, nanocomposites

## Abstract

Intelligent materials, such as memory shape polymers, have attracted considerable attention due to wide range of possible applications. Currently, intensive research is underway, in matters of obtaining memory shape materials that can be actuated via inductive methods, for example with help of magnetic field. In this work, an attempt was made to develop a new polymer composite—polyurethane modified with graphene nanoplates and ferromagnetic iron oxides—with improved mechanical properties and introduced magnetic and memory shape properties. Based on the conducted literature review, gathered data were compared to the results of similar materials. Obtained materials were tested for their thermal, rheological, mechanical and shape memory properties. Structure of both fillers and composites were also analyzed using various spectroscopic methods. The addition of fillers to the polyurethane matrix improved the mechanical and shape memory properties, without having a noticeable impact on thermal properties. As it was expected, the high content of fillers caused a significant change in viscosity of filled prepolymers (during the synthesis stage). Each of the studied composites showed better mechanical properties than the unmodified polyurethanes. The addition of magnetic particles introduced additional properties to the composite, which could significantly expand the functionality of the materials developed in this work.

## 1. Introduction

Memory shape materials are the center of growing attention as they offer a way to solve many problems in such a wide array of fields as aeronautics, medicine, transportation, electronics, design and various everyday products [1]. It is a group of intelligent materials that, when influenced by an external stimuli—heat, light, humidity, pH level, electric or magnetic field—can alter some of their physical parameters, usually shape, but also color or stiffness. Systems based on memory shape properties, unlike other intelligent systems, require no additional actuators. They react on their own to the change in their environment. Currently, the most researched group of materials in this category are polymers (SMP), as shape memory effect can be easily obtained—the only requirement is the presence of phase separation, such as in polyurethanes, acrylane copolymers and styrene based copolymers [1].

SMPs have many advantages, to name few: material properties can be fine-tuned to requirements via changes in materials, additives and synthesis method; SMP can be sensitive to different stimuli and with an adequate process the multiple shape memory effect can be achieved; high repeated deformation; low density; and possibility to modify the material by introducing fillers. Of course, they possess some disadvantages as well: their low stiffness that can influence the ability to maintain the temporary shape. Most of the synthesis methods require usage of organic solvents and limitations of the used polymer.

Currently, most of the existing SMP are thermally sensitive, however they are not a perfect solution to some applications, especially medical ones. In that area, magnetic shape memory polymers (MSMP) are usually used—patents already exist for smart catheter or intelligent implants for treating ocular hypertension [2]. They can also be used in systems monitoring drug delivery or implants that can be activated using magnetic field [3]. Thus far, inductive heating was reported for smart hydrogels, ferrogels and ferrofluids in an alternative magnetic field due to embedded magnetic particles [2]. MSMP materials can be used in vivo [4,5,6], if biocompatible materials that can be heated to ≤42 °C are used.

Other advantages of inductive heating include: easier construction (no need for power supply or cables nor for initiation mechanism) eliminating popular source of failure; elements can posses more complicated geometry, if the distribution of magnetic particles is even in the matrix; uniform heat propagation can be expected, no matter the elements geometry in its entire volume; if particles are distributed in specific sections, only these areas are transformed (at the same time the adhesion between various sectors is not impaired); and the stimuli can be delivered at a later point in time. Furthermore, majority of MSMP systems have similar shape memory level to thermally sensitive ones.

When describing magnetic shape memory effect, an additional parameter has to be included—field response time. That is the time value before T_trans_ is reached and when actual shape recovery process begins. It is especially important for elements used in medicine; for instance, it is imperative that recovery time of intravascular stents should be below 60 s [7]. In addition, the particle size can be of importance: Yu et al. proved that with rising diameter of iron oxide (Fe_3_O_4_) particles—from 10 to 100 nm—the shape recovery rate drops from 97% to just 20% [8].

Thermomagnetic or electromagnetic shape memory effect in nanocomposites can be reached by introducing particles of metal or their oxides as fillers, for example: iron oxide, neodymium magnet (NdFeB) particles, nickel powder (ZnNi) or ferromagnetic particles [1,2,9] (later referred to as *magnetic particles*). Magnetic properties are introduced to polymer composites usually by using micro- or nano-sized ZnNi or Fe_3_O_4_ particles [2]. However, due to magnetic properties and van der Waals forces, particles can agglomerate [10], therefore surface modification by surfactants or coupling agents, to promote polymer-magnetic bonding, is required. Particles of iron oxide are usually modified by use of silane coupling agents [11] or oleic layer [12] in order to decrease the amount of micro-sized agglomerates and ensure uniform dispersion. The impact of particle size on the energy loss and heating mechanism is complicated, but well described [13].

The induction mechanism can be described by the *Néel-Brown* relaxation model for one-domain ferromagnetic particles [2]. In the material, magnetic domains are formed with a fixed magnetization vector. The direction of the vector can change when the system is placed in a magnetic field. In an alternating magnetic current, the change in spins direction occurs constantly—this causes dissipation of the magnetic energy, which is transformed into thermal energy. Such losses are described by the *Néel-Brown model.* The *Néel* relaxation involves change only in the orientation of all the magnetic spins and not the physical rotation of the particle, while, in Brown relaxation, the spin is locked with the particle axis and the whole particle rotates [14]. The time of relaxation depends from the particle diameter—below 10 nm, the fast *Néel* relaxation dominates, while larger particles tend to follow the Brown model [15].

## 2. Experimental

As mentioned above, most SMP synthesis methods involve use of organic solvents—xylene [8], chloroform [16], DMF [17] or THF [18]—which are usually volatile and toxic. Although a green alternative in the form of water borne polyurethanes exists [19], both their mechanical and chemical properties are usually inferior and the possibility of phase separation between fillers and matrix exists. In this work, to manufacture the SMP materials, prepolymer method that does not require addition of organic solvents is used.

## 3. Materials

Polytetramethyleneglicole (PTMG, M = 2000 g/mol) was melted and degassed under vacuum at 75 °C. 4,4′-diphenylmethane diisocyante (MDI) was melted at 50 °C to separate dimmers from the rest of the phase. 1,4-butanediol (BDO) was dried under vacuum at 60 °C. Iron oxide particles (Fe_3_O_4_), provided by PyroGarage (Vilnius, Lithuania) with size ca. 45 μm were modified, while graphene nanoplatelets (GNP, size 2–10 nm) were used as obtained. 1,4-diazabicyclo[2.2.2]octane (DABCO) was used as catalyst; KH-550 (γ-aminopropyltriethoxysilan), ethanol (all materials provided by Sigma Aldrich, Poznań, Poland) and water were also used.

## 4. Modification of Fe_3_O_4_ Particles

Iron oxide particles were modified with KH-550 in accordance to the procedure described by Cai et al. [11]. In short, 15 g of Fe_3_O_4_ were dispersed in 500 mL of water-ethanol solution (1:1). Afterwards 8 mL of the compounding agent was added to the mixture and mixed for 4 h. The solution was left overnight for the sedimentation to occur. Next, the percipate was filtrated under reduced pressure and washed with the water-ethanol solution until neutral pH was obtained. Final participate [modified Fe_3_O_4_ (mFe_3_O_4_)] was dried under vacuum at 60 °C for 24 h.

## 5. Preparation of PU/Fe_3_O_4_/GNP Composites

For the prepolymer preparation, melted PTMG and MDI were mixed at 65 °C for 30 min and then secondly 85 °C for 2 h. Next, composites were synthesized by taking the prepolymer and mixing it with fillers for 60 s at high speed. Then, adequate amounts of BDO and DABCO were added. After mixing for 10 s, the mixture was poured out into hot forms which were consequently placed inside an oven for 24 h at 80 °C and then further incubated for 48 h at 60 °C. The overall ratio of PTMG/MDI/BDO was 1/4/1. Following Cai et al. [11], the Fe_3_O_4_ content was constant at 30 wt %, while the GNP content varied between 0 wt % and 1.2 wt %. The exact composition of the materials and their marking can be found in Table 1. The average thickness of materials was ca. 4 mm.

## 6. Characterization

Scanning Electron Microscopy (SEM) measurements of the fillers were conducted using the Quanta FEG 250 microscope (FEI Company, Hillsboro, OR, USA) with the ETD detector with 500–50,000× magnification at energy of 10 keV HV. Fourier transformation infrared spectra (FT-IR) were obtained using the Nicolet 8700 Spectrophotometer (Thermo Scientific, Madison, WI, USA with the measurement range of 500–4500 cm^−1^). For wide-angle X-ray scattering (XRD), Bragg-Brentano X’PERT PHILIPS diffractometer (PANalitical, Almelo, The Netherlands) was used to gather the XRD diffractograms (40 kV, 30 mA, λ Cu Kα = 0.1542 nm). Samples were scanned in 2θ range 5–80°. Differential Scanning Calorimetry (DSC) has been carried by DSC209F1 (NETZSCH, Selb, Germany) instrument in the temperature range from −85 °C to 250 °C. The samples weight was about 10 mg. Analysis was conducted in nitrogen atmosphere with flow 40 mL/min. The samples were pre-heated (1st-heating) to eliminate thermal history, then cooling and 2nd-heating at 20 °C/min. Dynamical Mechanical Analysis (DMA) has been carried by DMA Q800 analyzer (TA Instruments, New Castle, DE, USA. Temperature investigation mode has been performed in bending mode (single cantilever), in temperature range from −100 to 100 °C, 1 Hz frequency amplitude and heating rate 4 °C/min using liquid nitrogen as cooling medium. Thermogravimetry (TGA) of obtained materials was conducted by TG 209 F3 analyzer (NETZSCH, Selb, Germany). Degradation process has been performed in the temperature range from 35 °C to 600 °C, nitrogen atmosphere with flow rate 40 mL/min and heating rate 20 °C/min. The rheological properties of the filled prepolymers were investigated using rheometer R/S-CPS+ apparatus (AMETEK Brookfield, Middleboro, MA, USA) with the controlled shear rate (CSR) 100 or 300 s^−1^ at 70 °C. The mechanical properties were measured using the Static Testing Machine Z020 (Zwick/Roell, Ulm, Germany), with the stretching speed of 50 mm/min.

## 7. Shape Memory Measurements

The shape memory properties of the composites were measured using the common fold-deploy method [20], however because of poor results several changes were made trying to find the best programming process for the materials. This procedure contains three steps: (1) rectangular samples (90 × 10 × 1 mm) were heated to programming temperature (T_p_) in water bath and bent to a U-shape using a rod with 5 mm diameter to secure temporary shape; (2) samples were quickly placed into the ice-water bath for 5 min; and (3) samples were “released” and left for 5 min at rest temperature (T_l_)—the initial angle of the samples was recorded (θ_f_). Next, the samples were placed in the water bath at temperature of 45 °C and the change of shape was observed with time (t) meanwhile final angle of the sample (θ_r_) was being recorded. The procedure was repeated 3 times for each material, the temperature values were kept at constant value ±2 °C. The temporary shape retention rate (*R_f_*) and the shape recovery rate (*R_r_*) were calculated using the following formulas:

$$R_{f}=\frac{180{}^{\circ}-\theta_{f}}{180{}^{\circ}}\times 100\%$$

$$R_{r}=\frac{\theta_{r}}{180{}^{\circ}}\times 100\%$$

Due to low melting temperature of soft segments, partial shape recovery occurred when the material was left at the room temperature. This prompted the placement of samples at the lower temperature (+4 °C) and measurement of the initial change of shape (Δ_0_) while the material was “resting”:

$$\Delta_{0}=\frac{\theta_{f}-\theta_{0}}{\theta_{0}}\times 100\%$$

Thus far, to modify SMP carbon nanotubes (CNT, MWCNT) [21], graphene oxide [22], its reduced form [23] and their modifications were used—with only few articles referring solely to GNP. This is why one of the objectives of the research was to understand the influence of the temperature on such materials. Initial results could hardly be called promising, therefore more modifications to the programming were introduced (see Table 2). However, the basic procedure was left unchanged. To further investigate the influence of temperature on the sample, the heating of the material was performed at three temperatures (T_p_): 60, 80 and 90 °C. In other words, the shape memory investigation was performed under four different sets of conditions.

## 8. Results and Discussion

### Fillers Morphology

**Figure 1:**
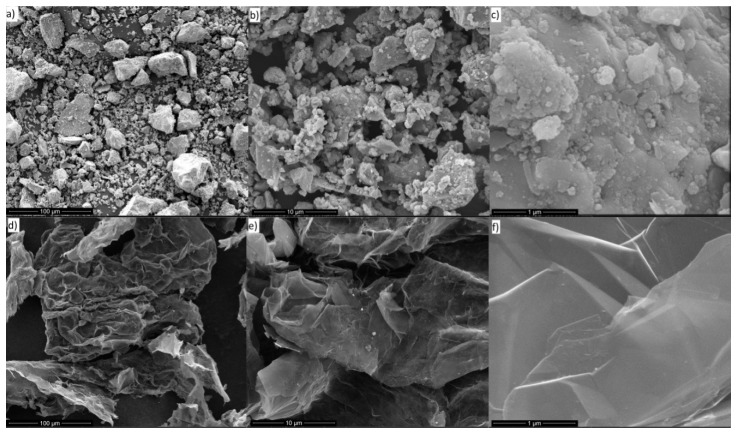
SEM images of: mFe_3_O_4_ (**a**–**c**); and GNP (**d**–**f**), at consecutive magnifications of 100, 10 and 1 μm.

Figure 1 shows the SEM analysis results of the used fillers. The Fe_3_O_4_ particles have average diameter of ca. 45 μm, but they can create lumps due to temporal auto-orientation of the magnetic domains. GNP are seen as thin flakes with diameters between 2 and 10 nm.

## 9. Spectroscopic Measurements

**Figure 2:**
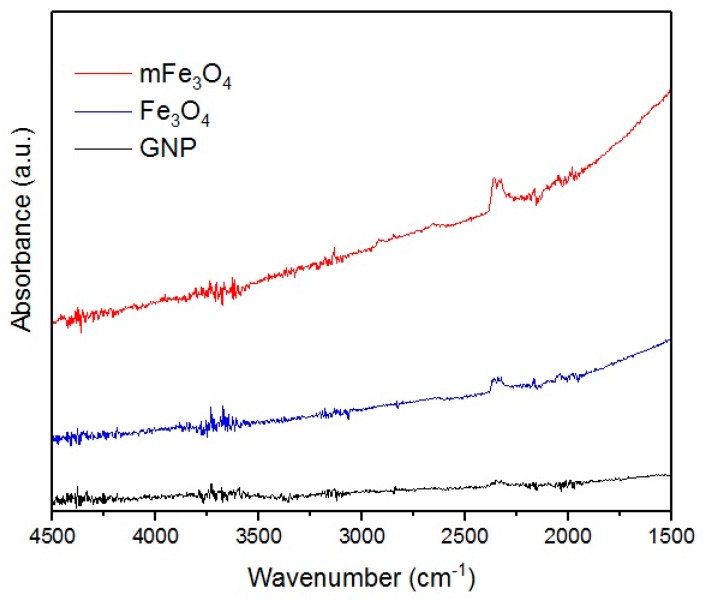
FT-IR spectra of used fillers.

**Figure 3:**
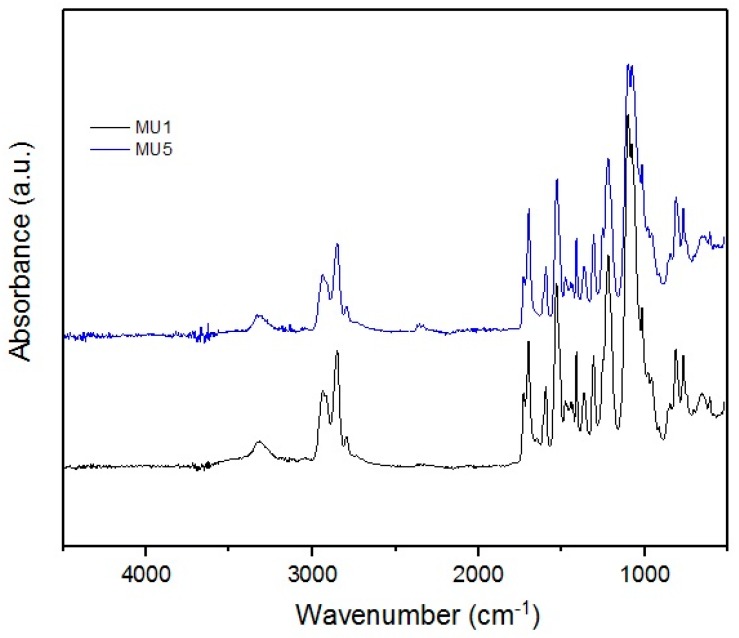
FT-IR spectra of MU1 and MU5 composites.

To analyze the structure of used fillers and obtained nanocomposites, FT-IR measurements were performed. Results are presented below on Figure 2 and Figure 3. Lack of any signals on the GNP spectra points towards the lack of functional groups, which can be related to results obtained by Geng et al. [24]. Fe_3_O_4_ and mFe_3_O_4_ particles were dried in vacuum directly before the measurements, therefore spectra do not contain signal at 3429 cm^−1^ corresponding to stretching vibrations of Fe–O–H (water absorbed on the particles surface) [25].

Obtained infrared absorption spectra of manufactured composites are very similar to each other—the synthesis did not include introduction of strong functional groups into the system. Characteristic signals for C–H stretching vibrations are visible at: 2828 and 2930 cm^−1^ (in the –CH_2_– group), and 1390 cm^−1^ (in the –CH_3_ group). At 1100 cm^−1^, the signal for stretching vibrations of C–O of ether group is found.

The presence of the urethane groups is the cause of the absorption signal at ca. 1700 cm^−1^, which corresponds to the stretching vibrations of C=O bond—this signal is also called the 1st amide signal, while the 2nd amide signal is located at 1530 cm^−1^, which is characteristic for deformative vibrations of N–H.

At 3300 cm^−1^, signals from secondary amides, i.e., N–H stretching vibrations, can be found. Due to the nature of the MU5 polyurethane, these signals can cover/envelope vibrations from KH 550 used to modify previously mentioned iron oxides—interactions with the filler could also cause slight shifts of this band towards lower wavenumbers.

In the MU5 composite, a small signal at 563 cm^−1^ is visible corresponding to the O=Fe–O bond in mFe_3_O_4_ particles. The bond is clearly visible in the filler spectra. The spectral range started at 500 cm^−1^, which is why the signals at low wave number are not very clear.

Analyzed FT-IR spectra clearly show that neither GNP nor mFe_3_O_4_ form new chemical bonds in polymer matrix—all changes in the composite properties are due to physical interactions between fillers and polymer chains [26,27].

**Figure 4:**
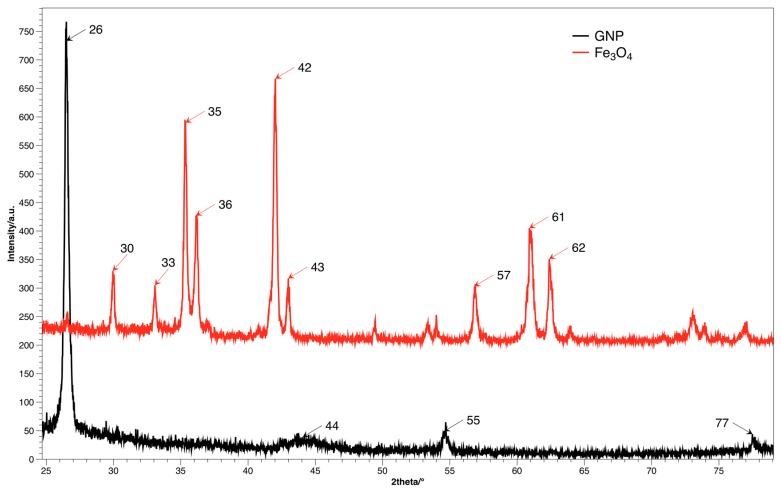
XRD diffractogram for fillers.

The crystal structure and size distribution was analyzed using the XRD method, spectra for both fillers are presented on Figure 4. The diffraction image of GNP contains the characteristic signal at shift 2θ = 26.4°, which corresponds to d_002_ plane, while distance between layers is ca. 3.4 Å [28]. Diffractogram of Fe_3_O_4_ shows signals for multiple planes (220), (311), (400), (511) and (440) for shifts 30°, 35°, 42°, 57° and 62° accordingly [16].

**Figure 5:**
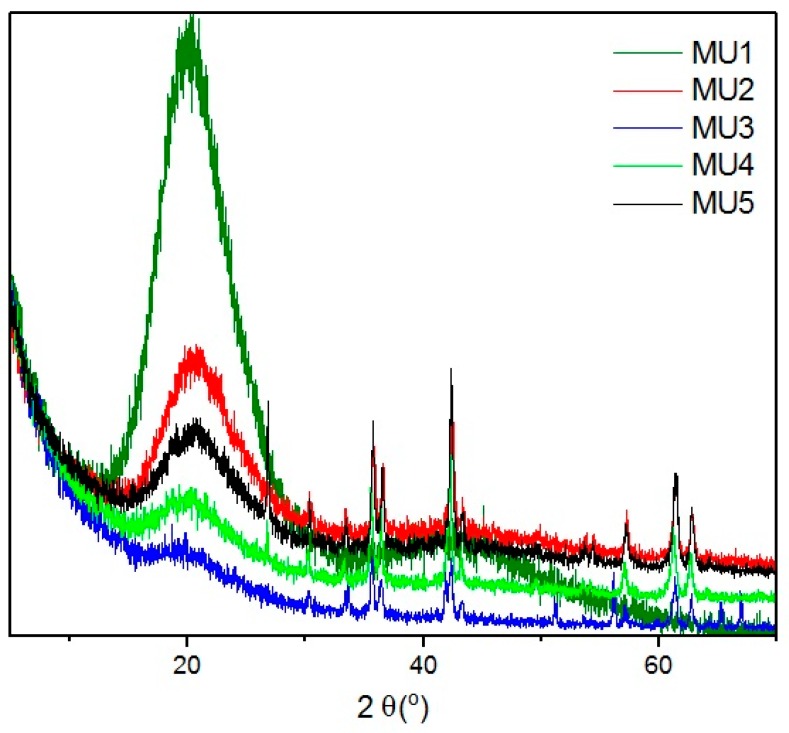
Diffractograms of different composites.

**Figure 6:**
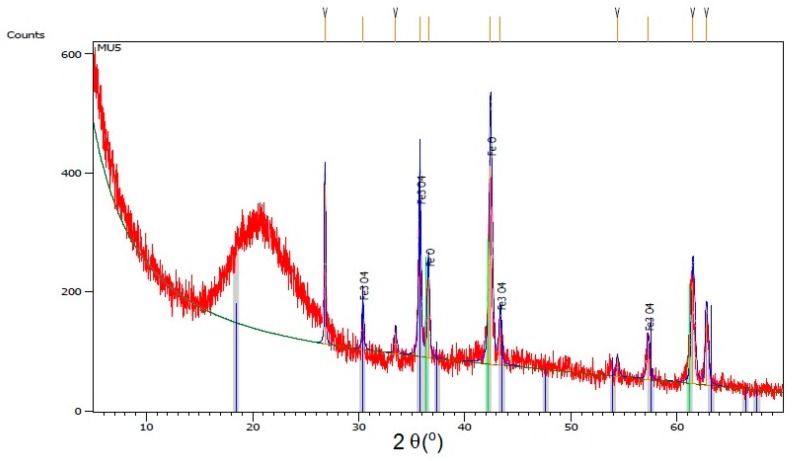
Isolated signals for mFe_3_O_4_ on the example of XRD diffractogram for the MU5 composite.

XRD spectra of all investigated composites (Figure 5) shows wide bands at angle shift 2θ = 20°, which can mean that polyurethane has mostly amorphous structure. At higher shifts, several diffraction maxima can be seen that can be attributed to mFe_3_O_4_ particles. Lack of significant bands for GNP can be assigned to its low content. Distinctive bands at shift 2θ = 26.6° and 54.7° for composites MU4 and MU5 can be attributed to GNP [26], which, in those two composites, is the most abundant. Lower intensity of the maxima (in comparison to GNP spectra) is a result of exfoliation of polymer matrix exfoliation. Shifts between layers in Fe_3_O_4_ and GNP particles are additionally specified on Figure 6, where XRD spectra for composite MU5 is presented

## 10. Thermal Investigation

**Figure 7:**
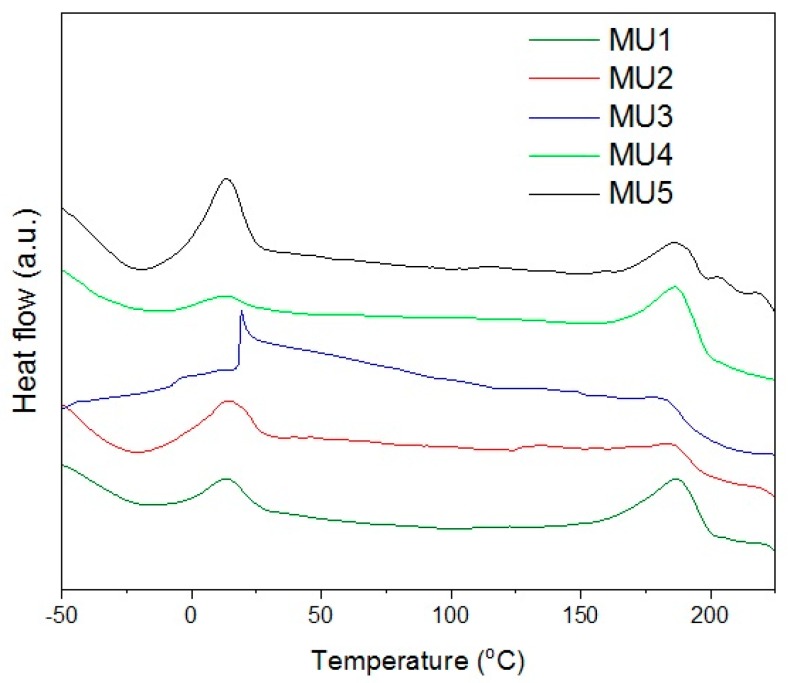
Second heating curve from the DCS.

The XRD analysis showed low degree of crystallinity in tested materials which is also visible on DSC thermograms—there are no maxima for changes in crystalline structure. Thus, it is not possible to use DSC as an accurate method verifying glass-transition temperature (it can be established from DMA measurements). Maxima visible on Figure 7 correspond to melting temperature of soft (SS) and hard segments (HS) in the polyurethane—temperature values are presented in Table 3. Additional analysis of surface area, below the maxima for hard segments melting range, allows estimating melt solid-liquid transition enthalpy.

Melting temperature of soft segments is relatively low (from 13 to 20 °C), which can be used to obtain the shape memory effect for composites. In order to fix temporary shape, the material has to be cooled down (and kept) below 10 °C. During storage, the temperature should not exceed Tmss, even if the programmed recovery temperature is higher.

**Figure 8:**
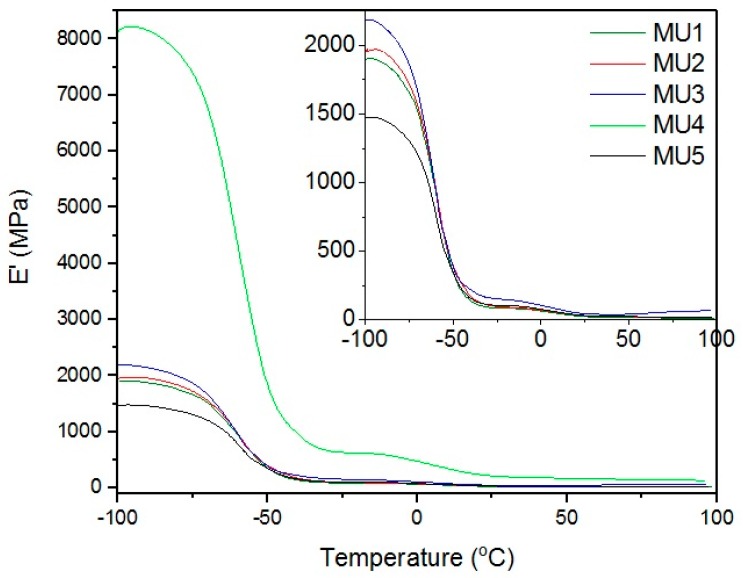
Relations between storage modulus E’ and temperature.

DMA results are compatible with the mechanical measurements, as described below (in Section 12). Results presented on Figure 8 show relations between storage modulus and temperature—the addition of filler improved mechanical properties of almost all of the composites. Drop in mechanical properties of MU5 can be attributed to the “oversaturation” with the filler. MU4 sample once again shows the best mechanical properties, where the storage modulus is over four times higher than for the unfilled polymer material.

**Figure 9:**
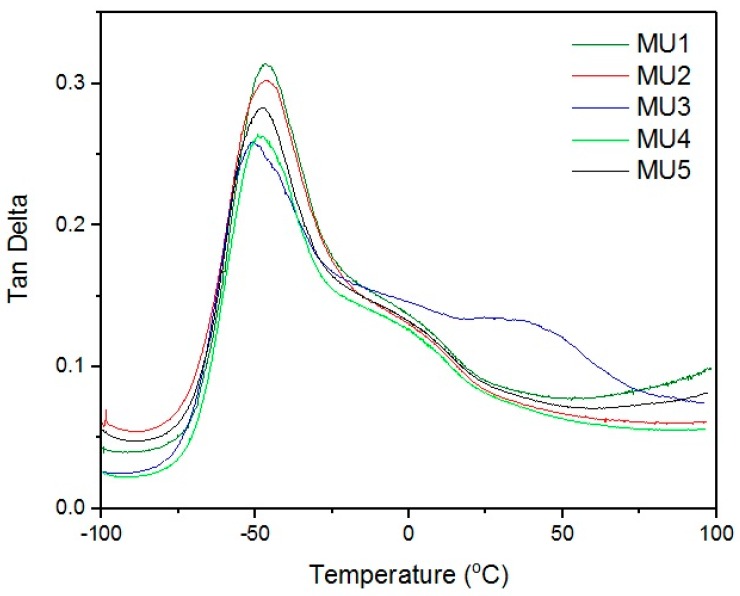
Relations between phase angle and temperature.

Maxima of phase angle relations (tan δ) and temperature (Figure 9) allowed the establishment of glass transition temperature of subsequent materials (results are summarized in Table 3). All of the tested materials have T_g_ value in a narrow range of −50.7 to −48.8 °C. Similar shape of all curves suggests close if not the same pattern of energy loss. At room temperature, materials are rather elastic, and their stiffness is imposed by the materials thickness.

**Figure 10:**
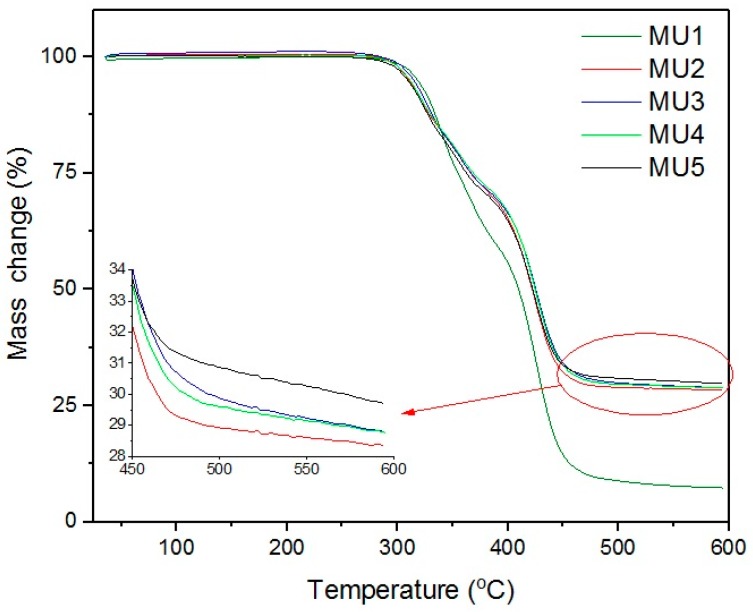
Thermogravimetric curves of all materials.

**Figure 11:**
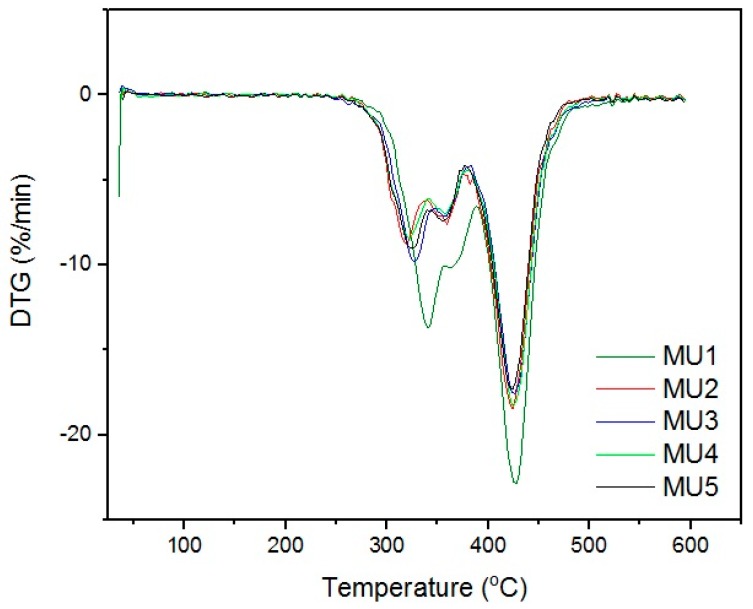
Differential thermogravimetric curves of all materials.

Thermogravimetric analysis results are presented on Figure 10 and Figure 11. The weight loss from temperature shows that the addition of fillers only slightly improves thermal stability of obtained composites, as the temperature values remain on similar level. The target content of fillers is at least 30%w, but weight loss in composites MU2–MU5 are higher, which points towards partial oxidation of the iron oxides.

Differential thermogravimetric analysis (DTG, Figure 11) shows that the decomposition of materials started around 320 °C. The double maxima on DTG curve were caused by decomposition of hard and elastic segments in polyurethanes. Minima around 425 °C could originate from two sources: degradation of aromatic isocyanates [29] or degradation of iron oxide particles in the coupling agent shell [30]. Mentioned phenomena are present in all of the samples, which indicates the source being the isocyanates or products of the first degradation. The temperature values of successive degradation stages (DTG curve minima) are summarized in Table 4.

Thermal properties of all examined materials do not show any significant differences between each other (Table 3), regardless of filler content. However, thermomechanical properties change slightly with the addition of GNP in comparison to iron oxides which do not have much influence in that matter.

## 11. Rheological Properties

**Figure 12:**
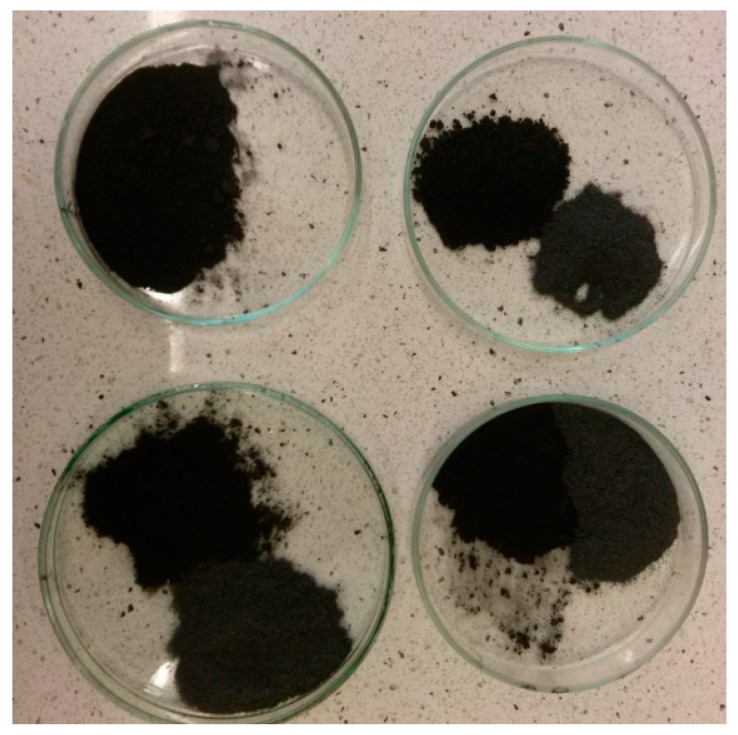
The amount of fillers added to adequate composites, from top left: MU2, MU3, MU4 and MU5.

Investigated materials contain over 30%w of the fillers in relation to overall prepolymer mass. However, added fillers have entirely different particle size—micro mFe_3_O_4_ and nano GNP. Therefore, the weight content can be deceiving in matters of discussing divergent bulk densities. That is why volume content could better illustrate the amount of fillers introduced to the system and the differences between each of them. In order to estimate the volume content (vt %) of fillers, the bulk density of all components had to be established. The bulk densities of prepolymer, mFe_3_O_4_ and GNP were 1.04, 2.00 and 0.06 g/cm^3^, respectively. Those values are however estimated and applicable only in this discussion and their purpose is to better illustrate fillers content. Both ways of showing different filler content in the material are presented in Figure 12 and Table 5.

**Figure 13:**
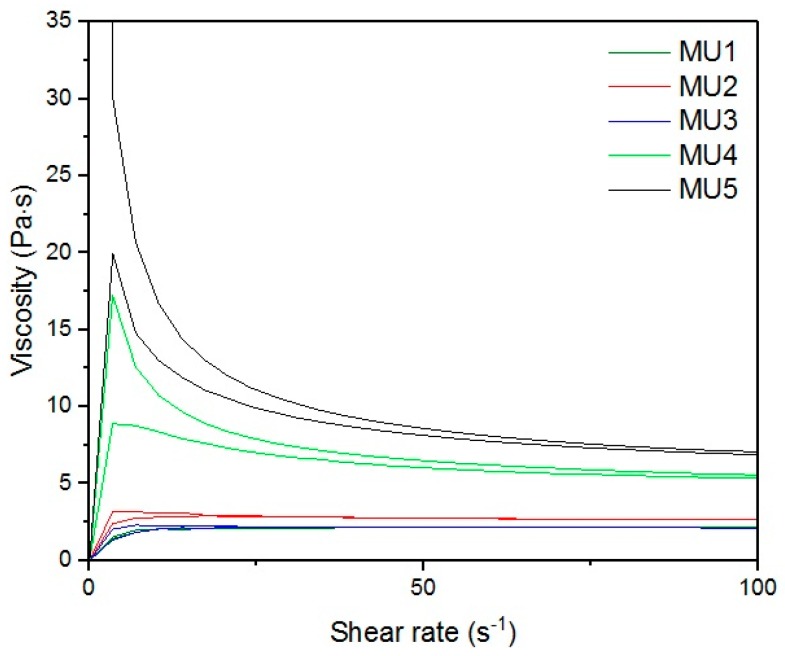
The viscosity-shear rate relations for all prepolymers at CSR = 100 s^−1^.

**Figure 14:**
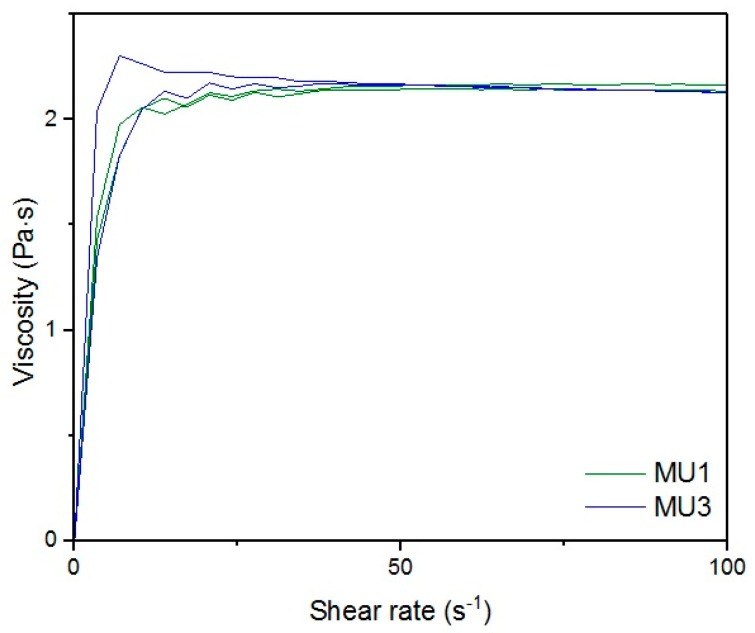
Magnification of the viscosity-shear rate relations for MU1 and MU3 prepolymers at CSR = 100 s^−1^.

**Figure 15:**
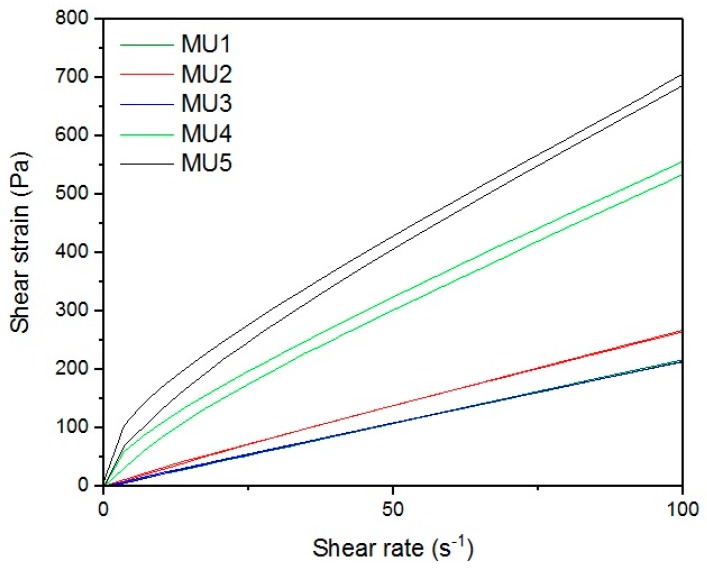
The shear strain-shear rate relations for all prepolymers at CSR = 100 s^−1^.

**Figure 16:**
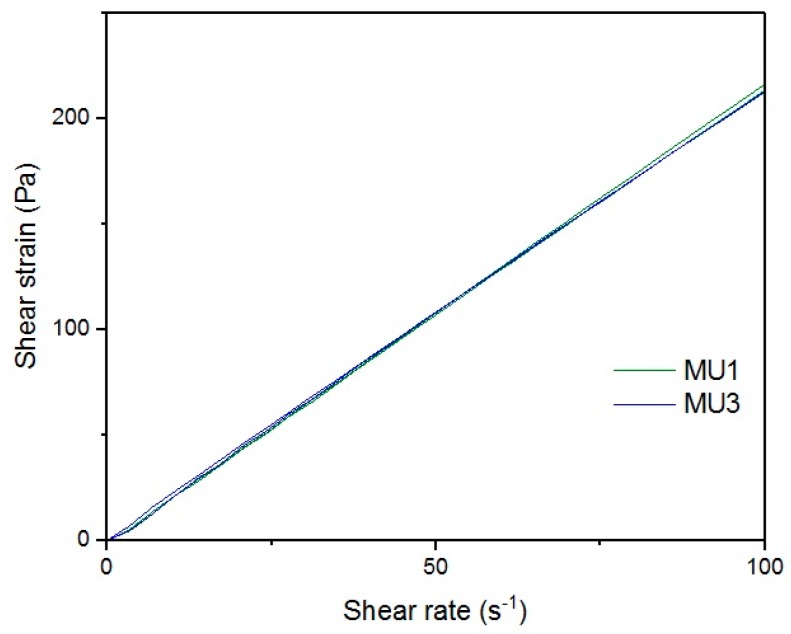
Magnification of the shear strain-shear rate relations for MU1 and MU3 prepolymers at CSR = 100 s^−1^.

**Figure 17:**
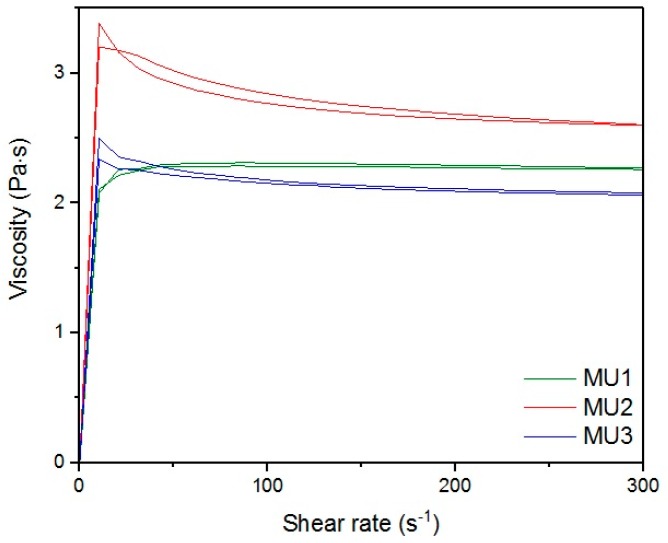
Magnification of the viscosity-shear rate relations for MU1 and MU3 prepolymers at CSR = 300 s^−1^.

**Figure 18:**
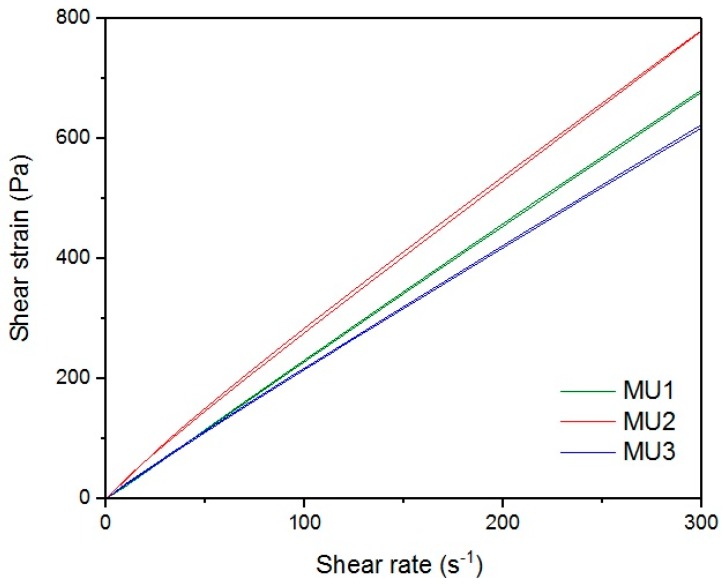
Magnification of the shear strain-shear rate relations for MU1–MU3 prepolymers at CSR = 300 s^−1^.

Figure 13, Figure 14, Figure 15, Figure 16, Figure 17 and Figure 18 show rheological measurements results of all prepolymers at 70 °C. Conducting the measurements for MU4 and MU5 prepolymers at higher shear rates proved impossible to conduct, as the stress values were too high. The measurements for MU1 and MU3 prepolymers were almost identical (Figure 14 and Figure 16, respectively). The higher the shear rate, the bigger the differences between analyzed prepolymers, but MU1 and MU3 are still very close to each other (Figure 17 and Figure 18, respectively).

**Figure 19:**
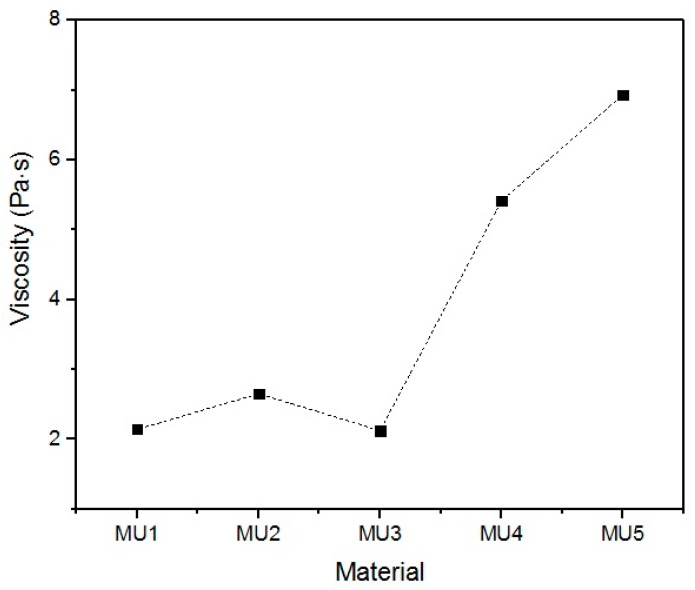
Influence of filler content on average prepolymer viscosity at CSR = 100 s^−1^.

**Figure 20:**
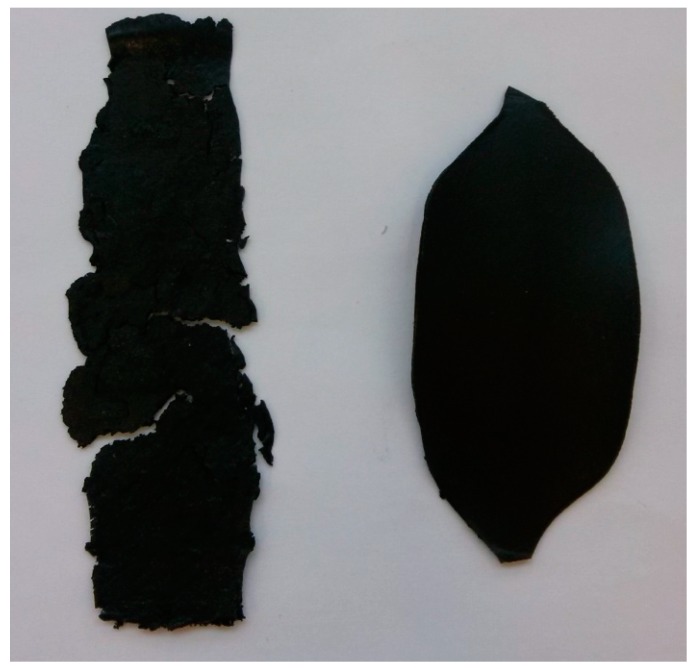
Materials MU2 (**left**) and MU3 (**right**) after pressing at same conditions.

With the increase in filler content, the average viscosity of the material increases, which is shown on Figure 19. It is not an entirely linear increase, furthermore the unfilled prepolymer has identical viscosity as the one with 20 vt %—they are both lower than the 13%v prepolymer. That difference was also seen while the materials were pressed during the shape memory tests. The MU2 composite was more problematic for thermal processing and the only one that required higher temperature and longer pressing, in order to obtain smooth surface (as seen in Figure 20 where both materials were pressed in the same conditions, however the surface appearance was significantly different). One can try to explain that in the following manner: the addition of iron oxide in MU2 impaired the polyurethanes flowability, but the consequent addition of GNP (MU3–MU5) improved thermal conductivity, which, in turn, enhanced the flowability of the system in higher temperatures (as in pressing). Nonetheless, this theory requires further investigation on that matter, for example if the effects can be seen as soon as temperature of 70 °C is reached.

High viscosity of filled prepolymers can prove quite an obstacle during composite production process, where the mixing stage is usually performed at higher shear rates. Again, that matter also needs further analysis—a good example of this could be change in mixing temperature.

## 12. Mechanical Properties

**Figure 21:**
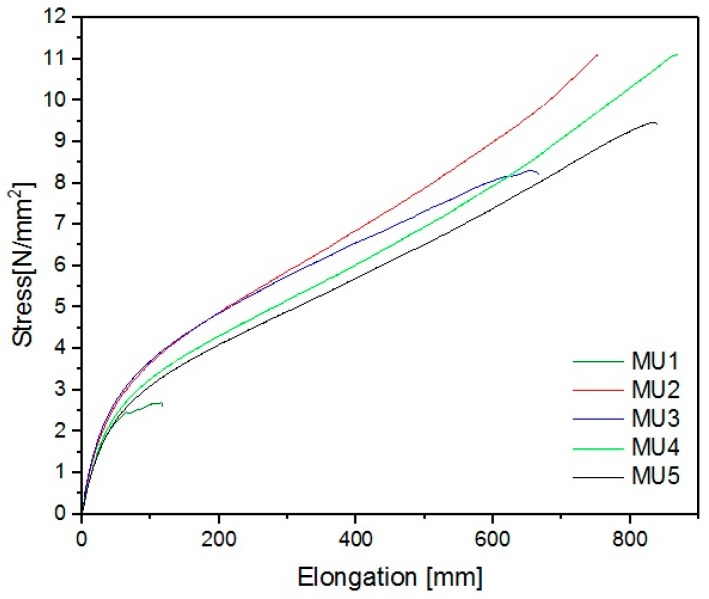
Elongation-stress curves for all composites obtained during stretching test.

When modifying polymer composites with powder fillers, the oversaturation effect of the material can sometimes be noticed—the amount added is so high that the properties of the material are heavily impaired. GNP is often used in polyurethane composites to improve the mechanical properties, while the main objective of mFe_3_O_4_ addition is to introduce new, unique properties—such as magnetic response from the material. Surprisingly, incorporating solely iron oxides enlarged material elongation at break parameter (Figure 21).

The best mechanical properties were achieved for the MU4 composite: breaking strain was 11 MPa and elongation 870 mm, which is several times higher than results for unmodified material—2.6 MPa and 117 mm, respectively. Despite a seemingly small difference between MU4 and MU5 (0.2%w/3.7%v in amount of fillers), signs of oversaturation in the material can be seen—breaking strain was 9.4 N/mm^2^ and elongation at break 840 mm. Young’s modulus of materials is low (in comparison to “standard” polyurethane results), however it was also improved by 80% for MU3 material. A summary of all parameters describing mechanical properties of the materials can be found in Table 6.

When analyzing results of this particular investigation it should be remembered that the materials were foamy. This could be the reason for low mechanical endurance, yet when analyzing breaking profiles, it was not determined that the break originated from air bubbles trapped in the polymer matrix.

## 13. Shape-Memory Properties

The shape memory properties were investigated under four different sets of conditions, as stated in the Table 2.

**Figure 22:**
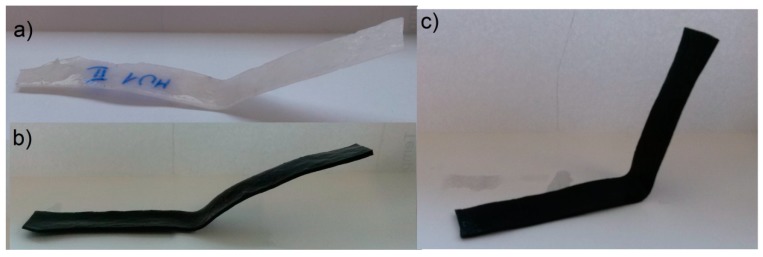
Angle of materials: (**a**) MU1; (**b**) MU3; and (**c**) MU5 right after the clamps were removed in the II set.

**Figure 23:**
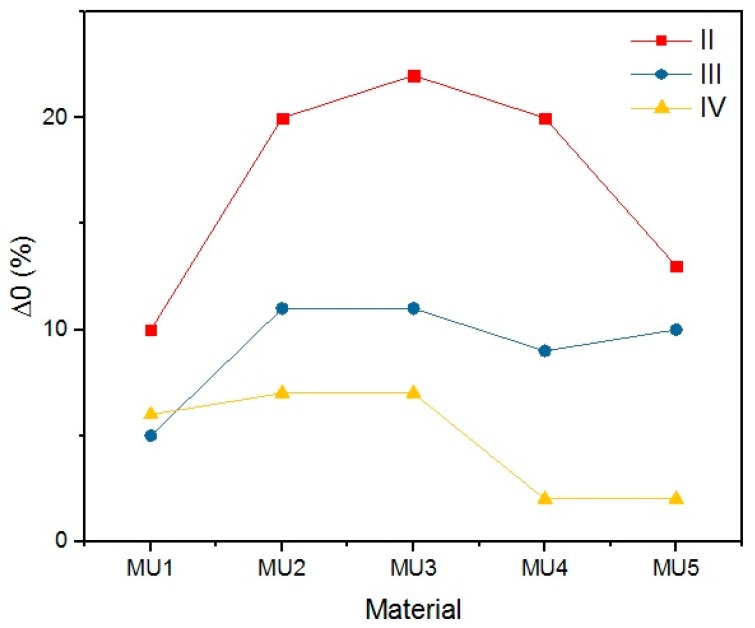
The shape change for materials that rested at 20 °C (I) or 4 °C (II–IV) which were programmed at different temperatures (I and II, 60 °C; III, 80 °C; and IV, 90 °C).

**Figure 24:**
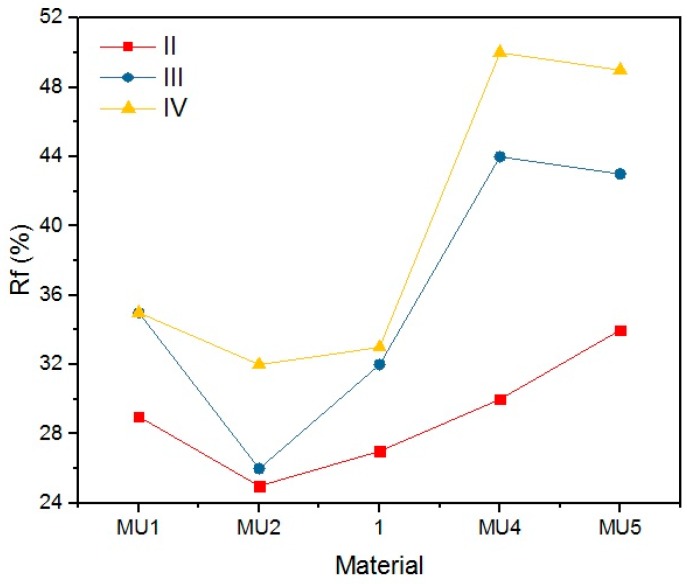
Shape retention rate after the rest period (T_l_ = 4 °C) for materials programmed at different temperatures (II, 60 °C; III, 80 °C; and IV, 90 °C).

**Figure 25:**
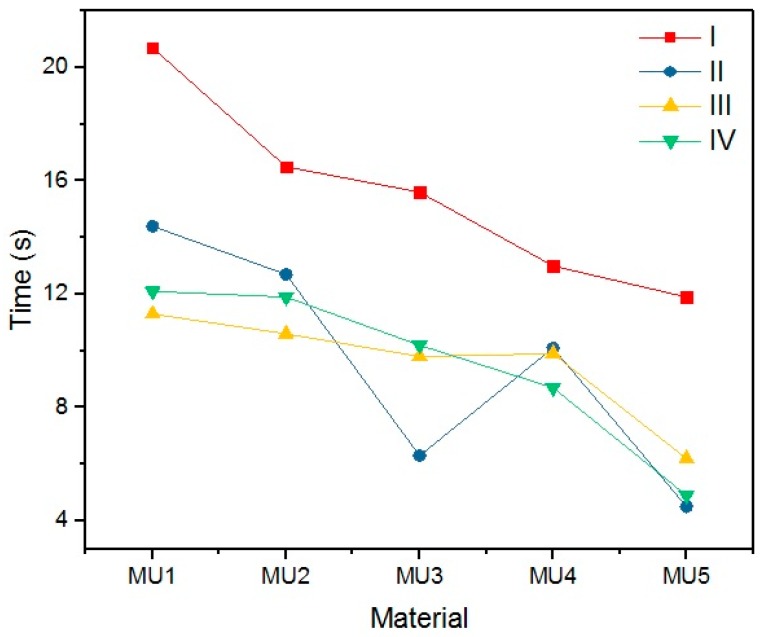
Time of the shape recovery process for materials programmed at different temperatures (I and II, 60 °C; III, 80 °C; and IV, 90 °C) and with different rest temperature (I, 20 °C; and II–IV, 4 °C).

**Figure 26:**
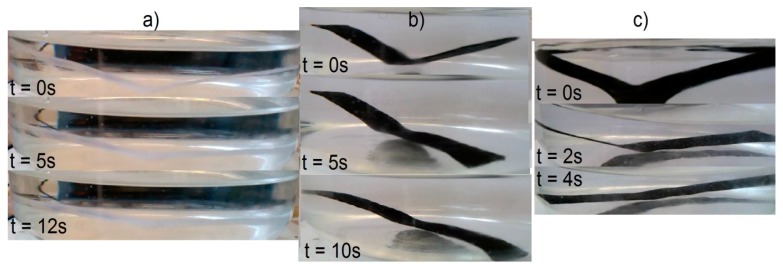
Shape recovery process for set III for materials: (**a**) MU1; (**b**) MU3 and (**c**) MU5.

The objective of the rest period is to check the fixation of the temporary shape. It is crucial not only to determine the storage conditions of such materials, but also the time while the temporary shape is still lasting. The change of shape during rest (Δ_0_) illustrates the fixation in time increments of 5 min. Even if a satisfactory deformation is obtained at the initial stage, it has to remain on the same (or at least similar) level for a given period of time. Differences in the initial shape between various composites is shown on Figure 22. Results of this investigation are demonstrated in Table 7, Table 8, Table 9 and Table 10 and Figure 23, Figure 24, Figure 25 and Figure 26.

Lower rest temperature has major influence on the shape retention during that time period, as the melt temperature of the soft segments is below the room temperature. Materials resting at 4 °C had lower Δ_0_ than materials programmed at the same temperature, but resting at 20 °C—the change was even three times lower, as seen in the MU5 material between the I and II set. Higher programming temperatures led to decrease of Δ_0_, which is more prominent in materials with higher filler content.

The materials were bent in half, yet the highest obtained deformation was 50% (90°) at T_p_ = 90 °C. The programming effectiveness seems to be dependent on temperature, as the highest chain mobility is achieved. The heating medium during the research was water, therefore the highest achievable temperature was 100 °C. It is possible that even higher programming temperature would lead to better deformation of the materials, however that hinders materials manipulation possibility. Table 8 and Figure 23 and Figure 24 present the shape retention rate after the rest period.

Materials presented a rather low shape retention rate (Rr). Furthermore, they spontaneously tried to regain their original shape (Δ_0_) during the rest period. The process was faster when the rest temperature of the material was higher than the melting temperature of the soft segments. The speed of shape recovery process increased exponentially after heating up the material. Low Rr is a result of high filler content, which disturbs the creation of segment structure, that allows creating the temporary shape of the material. Cai et al. [31] embodied MWCNT into the polymer chains (instead of a classic chain extender), and therefore they did not disturb the 3D structure of the chains, as they were incorporated. Although obtained results are not encouraging regarding material applications, it is possible that fine-tuning of the procedures could lead to better results.

R_r_ and Δ_0_ are dependent on three factors: the chemical composition, programming temperature and the rest temperature. It is linked to materials thermal properties—GNP addition increases thermal conductivity. With connection with high programming temperatures, sufficient chain mobility can be reached and the chains can be frozen in the temporary shape.

An important parameter while discussing SMPs is the time required for the material to recover its permanent shape after reaching T_trans_ (in this case 45 °C). Results of time measurements results are presented in Table 9 and illustrated on Figure 25.

Quite a surprise is the influence of the rest temperature on recovery time for materials programmed at the same temperature (sets I and II) which is between 20% and 60%. The addition of mFe_3_O_4_ had no noticeable influence on shape recovery, while 1.2% addition of GNP can reduce the time value by half. The programing temperature does not seem to have strong influence on the recovery time. However, the connection with T_p_ and R_f_ cannot be ignored, despite high R_f_ values for the first cycle, recovery time was noticeably higher. In addition, the temperature of shape recovery process can also influence the time value—when the recovery process was conducted at 50 °C, sample MU5 needed only 2.3 s for a full shape recovery. Example photos of the recovery process are presented on Figure 26.

Some important criteria when judging the possibility of materials application, is the ability to regain the original shape. After the recovery process, all materials regained the 180° angle, the lowest value was 178° at one attempt. The filler content seems to have no strong influence on the recovery ability of the material. Performing multiple cycles (15 repetitions, for set III) on one sample did not show any change in the shape memory behavior of the sample).

It is possible that such high recovery rate is a result of initial programming when samples were initially pressed. To check this theory further, investigation should be conducted by evaluating shape memory properties on a material that was not thermally processed. There is, however, a restriction regarding uniform (non-foamy), thick materials which could undergo previously mentioned programming procedure. Solvent casting could be used to obtain a thin film, however it requires the use of organic solvents.

In previous research, it is uncommon to find reference to SMPs that were initially pressed. However, references were found to PLA/GNP [28] and PCL/Fe_3_O_4_ [8] composites where the shape recovery rate was 83% and 80%, respectively. As the influence of pressing is nowhere evaluated, the differences cannot be attributed to the chosen fillers, as in all materials analyzed in this work (including the unfilled polyurethane matrix) have very similar, high level of R_r_. Another thermal processing method used for SMPs was the injection method, yet that reference source did not contain the R_f_ and R_r_ values [32].

**Figure 27:**
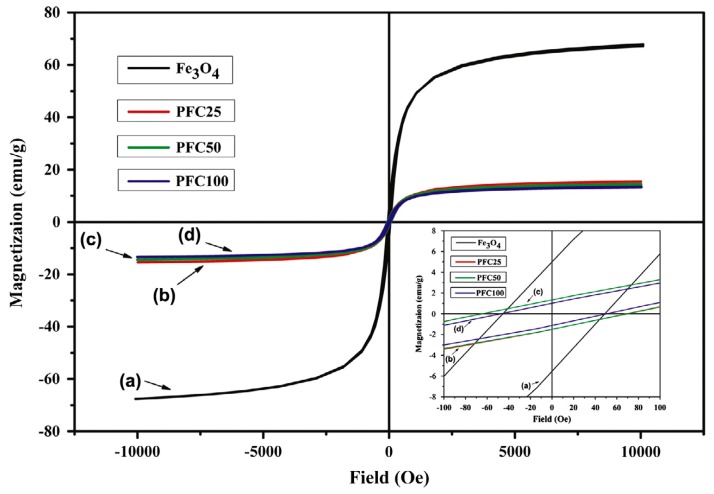
Magnetic hysteresis loop for Fe_3_O_4_ and PCL/MWCNT/Fe_3_O_4_ composites [31].

The next step would be to proceed with investigations regarding shape memory properties of composites placed in a magnetic field. As GNP shows no such properties, the magnetic response of these materials could resemble response of PCL/MWCNT/mFe_3_O_4_ composites, as seen on Figure 27.

Fe_3_O_4_ particles vibrations in the magnetic field are the source of thermal energy that leads to internal temperature increase up to T_trans_ value (45 °C). Therefore, the magnetic shape recovery investigation should include the response time for the internal temperature to increase up to the required value. Carbon nanofillers have good thermal conductivity, which provides quick internal heating of the material in its entire volume which speeds up the shape recovery process. Multiwall carbon nanotubes and graphene nanoplates have the same thermal conductivity—3000 W/mK [33], therefore the influence of both fillers on the composites heating rate should be the same. Table 11 contains comparison of materials shape memory properties (time and R_r_) programmed in the same conditions (T_p_ = 60 °C), unfortunately the source articles do not contain the data required here, i.e., respective R_f_ values. Polyurethane composites obtained in this research have higher shape recovery rate and lower response time.

## 14. Conclusions

After analyzing composite properties, we can notice a positive influence of added graphene nanoplates and ferromagnetic iron oxide.

Thermal properties of all composites remained on a similar level, however the mechanical properties increased significantly. Comparing to unfilled material (MU1), the breaking strain of composites MU4 and MU5 increased by 328% and 263%, respectively, while elongation at break increased by 641% and 616%.

With respect to memory shape properties, temperature trends described above cannot be ignored. At lower temperatures, the MU5 material had better retention rate (+17% compared to MU1), while, at higher temperatures, it increased by 43% and 40% for MU4 and MU5 materials, respectively. A seemingly small difference in their composition (0.2%w GNP) had high impact on time necessary for the shape recovery, it was 30%, or even 65% faster for MU4 and MU5 than for MU1 at the same temperature. However, discussing the “time” parameter, one has to bear in mind the actual difference between the temporary and permanent shape, as well as the desired application of such element. Presence of high strains during the shape recovery process might lead to micro cracks appearing in the material which could lower overall mechanical endurance.

In conclusion, a new, memory shape composite was successfully obtained. Fillers introduced to the polymer matrix lead to increase in some of the material properties, however, due to their powder nature, they might have disturbed overall segment structure inside the matrix.

Further investigation of this topic might include measurements in the magnetic field, while additional modifications of the composition and the programming process could lead to better results being obtained in near future.

## Figures and Tables

**Table 1:** The composition of the composites.

Name of the Composite	MU1	MU2	MU3	MU4	MU5
GNP content (wt %)	0	0	0.5	1.0	1.2
mFe_3_O_4_ content (wt %)	0	30	30	30	30
MU—Modified Urethane polymer					

**Table 2:** The overview of programming conditions for the shape memory investigation.

Set of Conditions	T_p_ (°C)	T_l_ (°C)
I	60	20
II	60	4
III	80	4
IV	90	4

**Table 3:** Results of thermal properties from DSC, DMA and TGA.

Material	Tg (°C) ^1^	Tm_SS_ (°C) ^2^	Tm_HS_ (°C) ^2^	ΔHm (J·g^−1^) ^2^	Weight Loss (%) ^3^
MU1	−46.9	13.4	186.4	3.40	92.7
MU2	−48.3	14.6	182.6	3.96	71.6
MU3	−50.6	19.2	180.1	2.14	71.2
MU4	−49.3	13.3	186.3	2.64	71.2
MU5	−48.0	13.9	185.9	1.16	70.3

^1^ Calculated from DMA measurements; ^2^ Calculated from DSC measurements; ^3^ Calculated from TGA measurements.

**Table 4:** Temperature values of successive degradation stages.

Material	T_1min_ (°C)	T_2min_ (°C)	T_3min_ (°C)
MU1	340	362	427
MU2	320	359	424
MU3	328	357	426
MU4	322	357	425
MU5	324	354	424

**Table 5:** The weight and volume amount of fillers in composites MU2–MU5.

Material	Filler	Weight Content (wt %)	Weight Content (wt %)	Volume Content (wt %)	Volume Content (vt %)	
MU2	mFe_3_O_4_	30	30.0	13.0	13.0
	GNP	0		0	
MU3	mFe_3_O_4_	30	30.5	13.0	22.3
	GNP	0.5		9.3	
MU4	mFe_3_O_4_	30	31.0	13.0	31.7
	GNP	1		18.7	
MU5	mFe_3_O_4_	30	31.2	13.0	35.4
	GNP	1.2		22.4	

**Table 6:** Summary of mechanical properties of the composites.

Material	Breaking Strain (MPa)	Elongation at Break		Young’s Modulus (MPa)
		(mm)	(%)	
MU1	2.6 ± 0.1	117	178 ± 9	0.0650 ± 0.0033
MU2	11.1 ± 0.6	753	602 ± 30	0.0855 ± 0.0043
MU3	8.2 ± 0.4	666	544 ± 27	0.1185 ± 0.0059
MU4	11.1 ± 0.6	870	680 ± 34	0.0861 ± 0.0043
MU5	9.4 ± 0.5	840	660 ± 33	0.0705 ± 0.0035

**Table 7:** The percentage value of shape change of the investigated materials (Δ_0_) during the rest period.

Nanocomposite	I	II	III	IV
MU1	23%	10%	5%	6%
MU2	27%	20%	11%	7%
MU3	49%	22%	11%	7%
MU4	44%	20%	9%	2%
MU5	39%	13%	10%	2%

**Table 8:** Percentage value of the shape retention rate (R_f_) after the rest period.

Material	I	II	III	IV
MU1	18%	29%	35%	35%
MU2	21%	25%	26%	32%
MU3	11%	27%	32%	33%
MU4	20%	30%	44%	50%
MU5	20%	34%	43%	49%

**Table 9:** Time of the permanent shape recovery after heating materials to 45 °C.

Nanocomposite	I	II	III	IV
	Time (s)			
MU1	20.7	14.4	11.3	12.1
MU2	16.5	12.7	10.6	11.9
MU3	15.6	6.3	9.8	10.2
MU4	13	10.1	9.9	8.7
MU5	11.9	4.5	6.2	4.9

**Table 10:** Permanent shape retention rate (R_r_) during the shape recovery process.

Nanocomposite	I	II	III	IV
	Retention Rate (%)			
MU1	100%	100%	99%	100%
MU2	100%	99%	100%	100%
MU3	99%	100%	99%	100%
MU4	100%	100%	100%	99%
MU5	100%	100%	100%	100%

**Table 11:** Memory shape properties at temperature 45 °C in water bath of materials obtained by Cai et al. [11,31] and materials obtained in this work according to the cycle II.

Properties	PCL/MWCNT/mFe_3_O_4_ Composite			PTMG/GNP/mFe_3_O_4_ Composite		
	CPF3	PCF50	PCF100	MU2	MU3	MU4
mFe_3_O_4_ content (wt %)	30	30	30	30	30	30
MWCNT/GNP content (wt %)	0	0.5	1	0	0.5	1
Time (s)	26.0	13.7	13.0	12.7	6.3	10.1
R_r_ (%]	90	95	93	100	99	100
